# Incidence of interstitial pneumonitis among breast cancer patients: a 10-year Danish population-based cohort study

**DOI:** 10.1038/sj.bjc.6604393

**Published:** 2008-05-27

**Authors:** S Christensen, L Pedersen, M Grijota, J B Kornum, A Beiderbeck, H T Sørensen

**Affiliations:** 1 Department of Clinical Epidemiology, Aarhus University Hospital, Aarhus, Denmark; 2 Worldwide Epidemiology, GlaxoSmithKline, Greenford, UK; 3 Department of Epidemiology, School of Public Health, Boston University, MA, USA

**Keywords:** interstitial pneumonitis, breast cancer, epidemiology

## Abstract

Chemotherapy and radiation therapy may increase risk for interstitial pneumonitis (IP) in breast cancer patients, but there are little current population-based data on IP incidence in these patients. We assessed population-based incidence rates (IRs) of IP among Danish breast cancer patients and compared these with IRs for the Danish general population. Through the Danish Cancer Registry, we identified all Danish breast cancer patients (*n*=35 823) diagnosed between 1994 and 2004. Treatment data were obtained from the Danish Breast Cancer Cooperation Group database, and data on IP, from the Danish National Registry of Patients. We computed IRs of IP among breast cancer patients and age-standardised incidence rate ratios (SIRs) comparing breast cancer patients with the general population. During follow-up, 28 breast cancer patients were registered with an IP diagnosis (IR=17.3 per 100 000 person-years (p-y) (95% confidence intervals (95% CI): 11.7–24.6)). When follow-up was restricted to 1 year after the first breast cancer diagnosis, eight patients with IP were identified (IR=23.4 per 100 000 p-y (95% CI: 11.0–44.1)). The SIR comparing breast cancer patients with the general population was 8.4 (95% CI: 5.7–11.9). Thus, although IP is a rare adverse event among breast cancer patients, its risk is substantially higher than that in the general population.

The improved prognosis and cure rates of breast cancer in recent decades underscore the need for data on chronic diseases and treatment sequelae (Cronin-Fenton *et al*, 2007). Interstitial lung diseases (ILDs) are a large and heterogeneous group of pulmonary fibrotic disorders, including interstitial pneumonitis (IP). Most cases of IP are of unknown cause (Camus *et al*, 2004; Raghu *et al*, 2004). Pulmonary drug toxicity is a common and possibly underdiagnosed cause of ILDs (Camus *et al*, 2004). It has been suggested that well-established breast cancer treatments, including tamoxifen and taxanes, increase the risk of IP, particularly when combined with adjuvant radiation therapy (Taghian *et al*, 2001; Yu *et al*, 2004; Burstein *et al*, 2006; Dimopoulou *et al*, 2006). Most of these studies of IP incidence among breast cancer patients are clinic-based case series without control groups, or reports based on data from clinical trials. Such trials often include small, highly selected study populations, limiting generalisability of their findings (Taghian *et al*, 2001; Yu *et al*, 2004; Burstein *et al*, 2006; Sorensen *et al*, 2006). Thus, it remains unclear whether, and to what extent, breast cancer is associated with later IP in a population-based setting. Owing to the low incidence of IP, studies of this condition generally require very large cohorts of breast cancer patients. Denmark's nationwide population-based health registries enable investigation of the incidence of this rare disease (Frank, 2000). In this population-based cohort study, we examined the incidence of IP among breast cancer patients and compared it with IP incidence in the Danish general population.

## MATERIALS AND METHODS

### Setting and design

We conducted this nationwide cohort study from 1 January 1994 to 31 December 2004, using data from Danish population-based health registries. The study's start date was chosen because on 1 January 1994 the Danish National Registry of Patients (NRP) replaced the eighth revision of the International Classification of Diseases (ICD) with the 10th revision for coding of the diagnoses. The 10th revision provides for more detailed coding of ILDs (Andersen *et al*, 1999).

The tax-funded National Health Service guarantees for all Danish residents access to health care, including free access to general practitioners, other primary medical care clinics, and all hospitals. In Denmark, public hospitals provide breast cancer patient care, including management of complications, such as IP, that arise during treatment.

Since 1968, the Danish Civil Registration System (CRS) has maintained electronic records for all Danish residents, including gender, date of birth, changes of address, dates of emigration, and changes in vital status (Frank, 2000). The 10-digit unique civil registration number assigned to every Danish resident is a unique personal identifier, enabling unambiguous linkage of records for this study from the NRP, the Danish Cancer Registry (DCR), the Danish Breast Cancer Cooperation Group (DBCG) database, and the CRS.

### The breast cancer cohort

We identified all first-time breast cancer patients (*n*=35 823) registered from 1994 to 2004 in the DCR (Jensen *et al*, 2002). The DCR is a population-based nationwide registry of all incident cases of cancer diagnosed in Denmark since 1943. The registry receives notifications of new diagnoses from hospital departments (including departments of pathology and forensic medicine), general practitioners, and practising specialists; the reported diagnoses are reclassified according to the modified seventh revision of International Classification of Diseases (ICD-7). For each case, the DCR also includes the patient's civil registration number, method of cancer verification, and tumour clinical stage according to the Summary Stage Classification. We used the following ICD-7 codes to identify breast cancer patients: 470.0–470.5, 870.0, 870.1, and 870.2. We excluded two patients who were registered with an IP diagnosis in the NRP before the date of their first breast cancer diagnosis. We grouped patients into four groups according to tumour stage at diagnosis (localised cancer only, regional metastases, distant metastases, and unknown).

The DBCG was established in 1976 with the purpose of collecting high-quality clinical, patient and treatment information on all breast cancer cases to ensure optimal diagnosis and treatment and to recruit patients to trials (Andersen and Mouridsen, 1988; Overgaard *et al*, 1997). Through the DBCG database, we obtained data on radiation therapy (breast alone or breast and axillary/neck), tamoxifen treatment, and chemotherapy for 22 748 (63.5%) breast cancer patients registered in DBCG between 1994 and 2004 and treated according to a prespecified protocol.

### Interstitial pneumonitis

The NRP contains data on all non-psychiatric hospital admissions in Denmark since 1977 and on all outpatient contacts, including ambulatory and emergency department visits, since 1995. Data include patients' civil registration numbers, dates of hospital admission and discharge, and up to 20 diagnoses (Andersen *et al*, 1999). We used the NRP to identify all hospital contacts during the 1994–2005 period for breast cancer patients registered with a primary or secondary diagnosis of IP associated with external agents (ICD-10 codes: J70.0-J70.9). We also used the NRP to obtain data on all diagnoses of IP in the general population registered between 1994 and 2005 inclusive.

### Validation of discharge diagnoses of IP

As hospital discharge diagnoses are not completely accurate (Sørensen, 1997), we evaluated the quality of the J.70.X diagnosis in the NRP by reviewing the hospital records of all patients registered with an IP diagnosis between 1994 and 2005 in North Jutland County (*n*=53). We confined the review to North Jutland County, which encompasses 10% of the entire Danish population, because of the uniform data quality in the county hospital discharge registries supplying data to the NRP.

Surgical lung biopsy is considered the gold standard for IP diagnosis (Hunninghake *et al*, 2001; Swigris *et al*, 2005), although the reproducibility of biopsy-proven ILDs has been shown to be low. In a clinical setting, lung biopsies are rarely performed because of potential complications, particularly prolonged pneumothorax in frail high-risk patients (Swigris *et al*, 2005). In the absence of biopsy data, we considered a discharge diagnosis of IP to be confirmed if the hospital record clearly stated that the patient had IP and if the diagnosis was not changed during further diagnostic work-up. We computed the positive predictive value (PV+) of an IP discharge diagnosis as the percentage of cases in the hospital record sample under review that fulfilled these criteria (Sorensen *et al*, 1996).

### Statistical analyses

We computed incidence rates (IR) of IP as the number of new IP cases per 100 000 person-years (p-y) of follow-up. Time at risk for breast cancer patients was computed as time from the first breast cancer diagnosis to the first IP diagnosis, death, emigration (obtained through linkage to the Danish CRS) or 31 December 2004, whichever came first. Then, we restricted follow-up time for IP to 1 year after the first breast cancer diagnosis. Time at risk for the general population was defined by the number of citizens alive in Denmark in the middle of the study period, that is, 1999 (obtained from Statistics Denmark). We repeated the analysis of IR for radiation-induced pneumonitis including patients treated with radiation therapy only.

We compared the number of observed IP cases among breast cancer patients with the number of expected IP cases in Denmark's general population by computing, as a measure of relative risk, standardised incidence ratios (SIR) as the ratio of the observed to the expected number of IP cases. Interstitial pneumonitis IRs per 100 000 p-y of follow-up by sex and age were computed for the general population. They were then applied to the p-y of observation for breast cancer patients to obtain the number of IP cases expected, if breast cancer patients had experienced the same IP rates as the general population. Ninety-five percent confidence intervals (95% CI) were computed for each SIR, assuming a Poisson distribution for the observed number of IP cases.

All statistical analyses were performed using SAS software (version 9.1.3, SAS Institute Inc., Cary, NC, USA).

## RESULTS

### Incidence of IP

**Figure 1:**
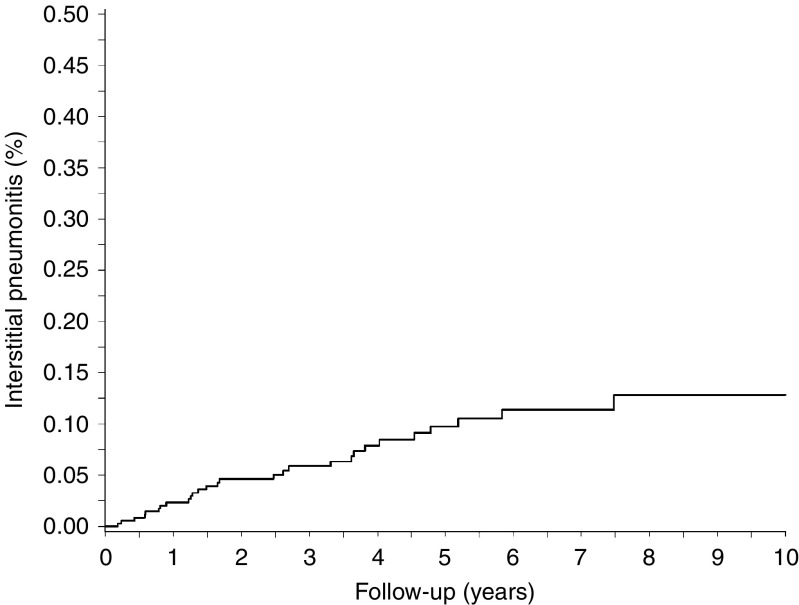
Cumulative incidence in percent of IP among 35 823 Danish breast cancer patients (1994–2004).

We identified 35 823 first-time breast cancer patients with a total follow-up time of 162 354 years (Table 1). The majority of breast cancer patients was between 50 and 70 years of age and most had regional metastases at time of diagnosis. Twenty-eight breast cancer patients were subsequently registered with a diagnosis of IP, corresponding to an IR of 17.3 (95% CI: 11.7–24.6) per 100 000 p-y (Figure 1). Twenty-four (85.7%) of the IP patients were only registered with one hospitalisation or outpatient contact for IP. Most IPs occurred among patients between 50 and 70 years of age (*n*=19, IR=22.6 (95% CI: 14.0–34.5) per 100 000 p-y). The majority of IP cases occurred in patients with regional metastases (*n*=18, IR=30.4 (95% CI: 18.6–46.9) per 100 000 p-y). The highest IR was among breast cancer patients with distant metastases (IR=60.7 (95% CI: 12.1–194.6) per 100 000 p-y), but this estimate, based on six cases, is statistically imprecise. With the exception of two IP cases recorded as drug-induced, the IPs were recorded as radiation-induced (92.6%). Eight cases of IP occurred within 1 year after the first breast cancer diagnosis, corresponding to an IR of 23.4 (95% CI: 11.0–44.0) per 100 000 p-y (Table 2). All these cases were documented as being radiation-induced.

Restricting the analysis to patients registered with complete treatment data in the DBCG database (*n*=22 748) (63.5%) and treated with radiation therapy (*n*=8090) left 14 cases of radiation-induced IP (IR=31.5 (95% CI: 18.5–51.5) per 100 000 p-y) (Table 3). With one exception, all cases were among patients treated with extensive radiation therapy (IR=56.9 (95% CI: 31.9–94.6) per 100 000 p-y); the remaining IP case was registered among patients treated with localised radiation therapy (IR=4.6 (95% CI: 0.4–21.6) per 100 000 p-y). The highest IR was found among patients treated with a combination of radiation and tamoxifen (IR=103.9 (95% CI: 46.3–204.5) per 100 000 p-y) or with a combination of radiation and chemotherapy (IR=42.5 (95% CI: 16.1–93.2) per 100 000 p-y). For patients treated with radiation, only the IR was 8.8 (95% CI: 1.7–28.1) per 100 000 p-y.

The overall SIR comparing IRs of IP among breast cancer patients with those in the general population was 8.4 (95% CI: 5.7–11.9), ranging from 28.9 (95% CI: 19.3–41.7) for radiation-induced IP to 1.5 (95% CI: 0.3–4.8) for drug-induced IP (Table 4).

### Validity of IP diagnoses

In the reviewed sample of 53 episodes of patients registered with IP, 41 episodes fulfilled criteria for confirmed IP, equivalent to a PV of 77% (95% CI: 63–89%). A total of 22 of 24 episodes registered as drug-induced IP fulfilled the criteria for IP (PV 92% (95% CI: 73–98%)) and 22 of 24 IPs registered as radiation-induced fulfilled our criteria (PV 87% (95% CI: 59–98%)). In all patients with a confirmed cancer diagnosis (14 of 53 patients), the IP diagnosis was considered correct. Most patients were diagnosed based on clinical and chest X-ray findings. Only four patients (7%) had lung-biopsy-confirmed diagnosis. High-resolution CT scans confirmed the diagnosis in eight patients (14%). The 12 patients with IP not confirmed by validation actual diagnoses included chronic obstructive pulmonary disease, asthma, congestive heart failure, or idiopathic pulmonary fibrosis.

## DISCUSSION

In a well-defined North European population, drug- or radiation therapy-induced IP requiring hospitalisation was a rare adverse event among breast cancer patients. However, the excess risk was substantial compared with that of the general population. Most cases of IP were considered radiation-induced and occurred more than 1 year following the initial breast cancer diagnosis. The highest IR was found among patients treated with radiation therapy and tamoxifen.

The strengths of our study include the uniformly organised Danish public health-care system, enabling a truly population-based design with little opportunity for diagnostic or referral bias. Further, we were able to include all patients with first-time hospitalisation for breast cancer; had access to detailed information on their cancer treatment; and had complete long-term follow-up for patients hospitalised with IP. The validity and completeness of breast cancer diagnosis recorded in the DCR has been shown to be very high (Jensen *et al*, 2002). The validity of our estimates thus depends ultimately on the quality of IP data in the Danish NRP, which is a function of diagnostic and coding practises. We found an acceptable positive predictive value of drug- and radiation-induced IP requiring hospitalisation, which was in the same range as for most other diseases reported in this registry (Sørensen, 1997).

Our study has several important limitations. Interstitial lung diseases are difficult to diagnose clinically (Raghu *et al*, 2004) and the validation procedure was hampered by the low proportion of patients undergoing surgical lung biopsy or high-resolution CT scans (Hunninghake *et al*, 2001; Cleverley *et al*, 2002). Our data lacked clinical detail and, as in other ILD registry-based studies, we were unable to apply the new classification of Diffuse Parenchymal Lung Disease (DPLD) to the ICD-10 diagnosis in the Danish NRP (Raghu *et al*, 2004). As this study aimed to examine adverse effects of breast cancer treatment, our focus was on DPLD cases caused by external agents such as drugs, including chemotherapy, and radiation therapy. The high SIR for IP among breast cancer patients should be interpreted with caution because radiation-induced IP was defined in terms of the exposure, that is radiation therapy (Rothman, 2002), and because the majority of persons in the general population were not treated with radiation therapy and they were therefore not at risk for radiation-induced IP. Still, the SIR allowed us to quantify the excess IP risk among breast cancer patients.

In addition, by using hospital diagnoses to identify IP cases, we may have missed patients with few or mild symptoms of IP. Consequently, the IP IRs observed in our study must be viewed as conservative estimates of the true IRs. Still, inclusion of outpatient data in the NRP from 1995 onwards increased the sensitivity of the data by reducing underreporting of mild IP. We lacked data on severity of IP symptoms, and the number of hospitalisations may be a poor proxy measure of severity because patients with severe IP may die soon after first IP hospitalisation.

Compared with the general population, breast cancer patients are in closer and more regular contact with the health-care system, so that greater observed risk of IP among them may be partly explained by surveillance bias. On the other hand, it is also possible that diagnostic neglect of end-stage breast cancer patients in the health-care system led to underestimation of IP incidence in this group.

Despite the limitations discussed above, the IP IRs we identified in the general population were in the same range as those reported by the only two other population-based studies on this condition, conducted in New Mexico, USA, and Southern Spain (Coultas *et al*, 1994; Lopez-Campos and Rodriguez-Becerra, 2004). This provides confirmation for the methodological strength of our study.

Despite the availability of complete nationwide follow-up for IP among all breast cancer patients during the 10-year study period, the number of observed IP cases was small, and it is difficult to interpret the resulting imprecise estimates. As chemotherapy and radiation therapy, and particularly the combination of the two, increase the risk of IP, we expected the incidence of IP among breast cancer patients to be higher than in the general population (Taghian *et al*, 2001; Burstein *et al*, 2006). However, there are no population-based data on the magnitude of this increased risk. In two recent US randomised controlled trials examining the risk of pneumonitis among breast cancer patients treated with taxanes, the proportion of patients developing pneumonitis was as high as 15% (Taghian *et al*, 2001; Burstein *et al*, 2006). However, generalisation of these results to all patients with breast cancer is complicated by the fact that these populations are highly selected and closely monitored (Sorensen *et al*, 2006). Our findings of the highest IR of IP among patients treated with radiation therapy and tamoxifen is in line with previous findings, suggesting that tamoxifen mediates the enhancement of radiation-induced lung fibrosis, possibly by inducing transforming growth factor-β secretion (Bentzen *et al*, 1996).

Cancer treatment is the suggested mechanism underlying the association between cancer and IP. Since lung tissue is included in radiotherapy of breast cancer, these patients have a higher risk of radiation-induced IP than patients with other solid tumours. This is corroborated by our findings of a much higher IR of IP among patients with documented radiation therapy and by greater IRs seen among breast cancer patients treated with extensive *vs* localised radiation. It is, therefore, questionable whether the IRs of IP in our study can be validly generalised to other cancer patients.

In conclusion, IP is a rare adverse event among breast cancer patients, but the risk is substantially higher than that in the general population.

## Figures and Tables

**Table 1:** Incidence of interstitial pneumonitis (IP) among 35 823 Danish breast cancer patients by age and cancer stage, Denmark 1994–2004

	**Number of breast cancer patients (%)**	**Number of IP patients (%)**	**Incidence rate per 100 000 person-years (95% CI)**
*IP unspecified*			
Overall	35 823 (100%)	28 (100%)	17.3 (11.7–24.6)
*Age groups (years)*			
<50	7119 (19.9%)	6 (21.4%)	16.4 (6.8–33.7)
50–70	17 694 (49.4%)	19 (67.9%)	22.6 (14.0–34.5)
>70	11 010 (30.7%)	3 (10.7%)	7.3 (2.1–19.3)
*Cancer stage*^a^			
Localised cancer, no metastasis	13 746 (38.4%)	2 (7.1%)	6.9 (2.9–14.3)
Regional metastases	16 841 (47.0%)	18 (64.3%)	30.4 (12.6–46.9)
Distant metastases	1881 (5.2%)	6 (21.4%)	60.7 (12.1–194.6)
Unknown	3355 (9.4%)	2 (7.1%)	14.8 (2.9–47.6)
			
*IP radiation-induced*			
Overall	35 823 (100%)	26 (100%)	16.0 (10.7–23.1)
*Age groups (years)*			
<50	7119 (19.9%)	6 (23.1%)	16.4 (6.8–33.7)
50–70	17 694 (49.4%)	17 (65.4%)	20.7 (12.2–31.6)
>70	11 010 (30.7%)	3 (11.5%)	7.3 (2.0–19.3)
*Cancer stage*^a^			
Localised cancer, no metastasis	13 746 (38.4%)	2 (7.7%)	4.6 (1.6–11.0)
Regional metastases	16 841 (47.0%)	18 (69.2%)	60.7 (12.1–194.6)
Distant metastases	1881 (5.2%)	2 (7.7%)	30.4 (18.6–46.9)
Unknown	3355 (9.4%)	2 (7.7%)	14.8 (2.9–47.6)
			
*IP drug-induced*			
Overall	35 823 (100%)	2 (100%)	1.2 (0.25–3.9)
*Age groups (years)*			
<50	7119 (19.9%)	0	—
50–70	17 694 (49.4%)	2 (100%)	2.4 (0.5–7.6)
>70	11 010 (30.7%)	0	—
*Cancer stage*^a^			
Localised cancer, no metastasis	13 746 (38.4%)	0	—
Regional metastases	16 841 (47.0%)	0	—
Distant metastases	1881 (5.2%)	2 (100%)	2.3 (0.5–7.4)
Unknown	3355 (9.4%)	0	—

a Cancer stage according to summary stage classification.

**Table 2:** Incidence of interstitial pneumonitis (IP) cases registered within one year following initial breast cancer diagnosis, by age and cancer stage, Denmark 1994–2004

	**Number of breast cancer patients (%)**	**Number of IP patients (%)**	**Incidence rate per 100 000 person-years (95% CI)**
*IP unspecified*			
Overall	35 823 (100%)	8 (100%)	23.4 (11.0–44.0)
*Age groups (years)*			
<50	7119 (19.9%)	2 (25.0%)	28.4 (5.7–91.1)
50–70	17 694 (49.4%)	6 (75.0%)	34.9 (14.5–71.8)
>70	11 010 (30.7%)	0	—
*Cancer stage*^a^			
Localised cancer, no metastasis	13 746 (38.4%)	0	—
Regional metastases	16 841 (47.0%)	7 (87.5%)	52.2 (23.3–102.5)
Distant metastases	1881 (5.2%)	0	—
Unknown	3355 (9.4%)	1 (12.5%)	33.1 (3.0–154.5)
			
*IP radiation-induced*			
Overall	35 823 (100%)	8 (100%)	23.4 (11.0–44.1)
*Age groups (years)*			
<50	7119 (19.9%)	2 (25.0%)	28.4 (5.7–91.1)
50–70	17 694 (49.4%)	6 (75.0%)	34.9 (14.5–71.9)
>70	11 010 (30.7%)	0	—
*Cancer stage*^a^			
Localised cancer, no metastasis	13 746 (38.4%)	0	—
Regional metastases	16 841 (47.0%)	7 (87.5%)	52.2 (23.3–102.5)
Distant metastases	1881 (5.2%)	0	—
Unknown	3355 (9.4%)	1 (12.5%)	33.1 (3.0–154.5)
			
*IP drug-induced*			
Overall	35 823 (100%)	0	—

a Cancer stage according to summary stage classification.

**Table 3:** Incidence of interstitial pneumonitis cases among 8090 breast cancer patients registered in DBCG^a^ and treated with radiation therapy by age and cancer stage, Denmark 1994–2004

	**Number of breast cancer patients (%)**	**Number of IP patients (%)**	**Incidence rate per 100 000 person-years (95% CI)**
*IP radiation-induced*			
Overall	8090 (100%)	14 (100%)	31.5 (18.1–51.5)
*Age groups (years)*			
<50	2971 (36.7%)	2 (14.3%)	11.7 (2.3–37.4)
50–70	4577 (56.6%)	10 (71.4%)	40.5 (20.8–71.8)
>70	542 (6.7%)	2 (14.3%)	7.3 (1.6–26.0)
*Cancer stage*^a^			
Localised cancer, no metastasis	3444 (42.6%)	1 (7.1%)	4.7 (0.4–22.0)
Regional metastases	4311 (53.2%)	13 (92.9%)	61.1 (34.2–101.5)
Distant metastases	93 (1.1%)	0 (−)	—
Unknown	242 (3.0%)	0 (−)	—

a Danish Breast Cancer Corporation Group database.

**Table 4:** Age-standardised incidence ratios (SIRs) of interstitial pneumonitis among 35 823 Danish breast cancer patients diagnosed between 1994 and 2004

	**Observed number of IP cases**	**Expected number of IP cases**	**SIR (O/E) (95% CI)**
All interstitial pneumonitis	28	3.34	8.4 (5.7–11.9)
Radiation-induced interstitial pneumonitis	26	0.90	28.9 (19.3–41.7)
Drug-induced interstitial pneumonitis	2	1.34	1.5 (0.3–4.8)
